# Long‐Term Opioids in Gout: A Matched Cohort Study From the Veterans Health Administration

**DOI:** 10.1002/acr.25622

**Published:** 2025-11-27

**Authors:** Lindsay N. Helget, Bryant R. England, Punyasha Roul, Harlan Sayles, Tuhina Neogi, James R. O'Dell, Joshua F. Baker, Ted R. Mikuls

**Affiliations:** ^1^ Veterans Affairs Nebraska‐Western Iowa Health Care System and University of Nebraska Medical Center Omaha; ^2^ University of Nebraska Medical Center Omaha; ^3^ Boston University School of Medicine Boston Massachusetts; ^4^ Corporal Michael J. Crescenz Veterans Affairs Medical Center and University of Pennsylvania Philadelphia

## Abstract

**Objective:**

Though used frequently to treat flare, risk of long‐term opioid exposure in gout has not been well defined. In this study, we examined the hypothesis that people with gout are more likely than individuals without gout to be prescribed long‐term opioids over time.

**Methods:**

In this matched cohort study using national Veterans Health Administration (VHA) data, multivariable Cox regression was used to examine the association of gout with long‐term opioid receipt (defined using a validated administrative algorithm). Patients with gout were identified using diagnostic codes and matched with up to 10 controls without gout by age, sex, and VHA enrollment year. In analyses limited to gout, factors associated with long‐term opioid exposure were identified.

**Results:**

Over a mean follow‐up of 4.52 years, patients with gout were more likely to receive long‐term opioids than controls (6.9% vs 3.8%). This risk remained following covariate adjustment (adjusted hazard ratio 1.30; 95% confidence interval 1.28–1.32). Among patients with gout, factors independently associated with an increased likelihood of long‐term opioid exposure included more recent index year, younger age, female sex, non‐Hispanic Black race and ethnicity, rural residence, being underweight or obese, former or current smoking, greater comorbidity, urate‐lowering therapy receipt, and requirement of rheumatology consultation.

**Conclusion:**

Patients with gout are more likely to receive long‐term opioids than counterparts without gout, independent of other factors. This risk is greater in underrepresented gout populations, those with greater comorbidity, patients requiring rheumatology consultations, and individuals prescribed urate‐lowering treatments. Additional investigation is needed to elucidate whether deployment of optimal gout management minimizes long‐term opioid exposure in patients with gout.

## INTRODUCTION

Characterized by recurrent and often severely painful articular flares, gout represents the most common form of inflammatory arthritis worldwide.^1^ National prevalence estimates approach 4%, which translates to >10 million individuals with gout in the United States alone.^2^, ^3^, ^4^ The burden posed by gout is even greater among select populations, such as those receiving care in the US Veterans Health Administration (VHA), where its prevalence approaches 6%.^2^ The articular deposition of monosodium urate leading to arthritis flares, though potentially preventable with appropriate management, frequently results in progressive joint damage, disability, and marked pain. Consequently, gout leads to substantial health care use, including >200,000 attributable emergency department encounters annually in the United States and rates of inpatient hospitalizations for gout doubling between 1993 and 2011.^5^ Gout‐related health care encounters are frequently accompanied by prescriptions for anti‐inflammatory medications and other analgesics, including opioids.^6^ Previous surveys have shown that between 20% and 30% of patients with gout receive an opioid prescription at the time of discharge from an emergency department encounter.^7^, ^8^

**SIGNIFICANCE & INNOVATIONS**

- This is among the first studies to evaluate long‐term opioid use in a US gout population.
- In the Veterans Health Administration (VHA) from 2000 to 2020, patients with gout were 30% more likely to receive long‐term opioid therapy, independent of other factors.
- Factors most strongly associated with an increased likelihood of long‐term opioid exposure in patients with gout included female sex, underweight body mass index (BMI), current smoking, a higher Rheumatic Disease Comorbidity Index score, urate‐lowering therapy receipt, and requirement of rheumatology consultation.
- Factors associated with a lower risk of long‐term opioid exposure in patients with gout included overweight BMI, the presence of chronic kidney disease, and evidence of adequate serum urate control.

The United States continues amid an opioid epidemic that has accounted for >100,000 deaths in 2021 alone, twice the number of opioid‐related US deaths in 2015.^9^ With similar trends in other parts of the world, approximately one‐third of deaths from opioid overdose are ascribed to misuse of prescription medications.^10^ Examining claims data, Brummett and colleagues observed that among patients receiving postprocedure opioid analgesics, up to 6% transitioned to long‐term use.^11^ Indeed, opioid prescriptions for acute‐pain management are well recognized to serve as a “gateway” to long‐term use.^12^ With reports of frequent administration and dispensing in short‐term management settings, it remains unknown whether gout might similarly serve as a risk factor for long‐term opioid exposure.

To date, there have been no large‐scale investigations examining the frequency of long‐term opioid use in gout. In this study, we sought to examine the long‐term risk of prolonged opioid exposure in gout, testing the hypothesis that gout would predispose patients to a higher risk of exposure to long‐term prescription opioids compared to patients without gout. Among patients with gout, we also sought to identify factors associated with long‐term opioid exposure over time.

## METHODS

### Study design and participants

We performed a matched cohort study within the VHA. We examined national Veterans Affairs (VA) administrative and electronic health record data from January 2000 through July 2020, accessed through the VA Corporate Data Warehouse (CDW) located within the VA Informatics and Computing Infrastructure.^13^ The study was approved by the VA Nebraska‐Western Iowa Health Care System Institutional Review Board with a waiver of informed consent.

**Figure 1:**
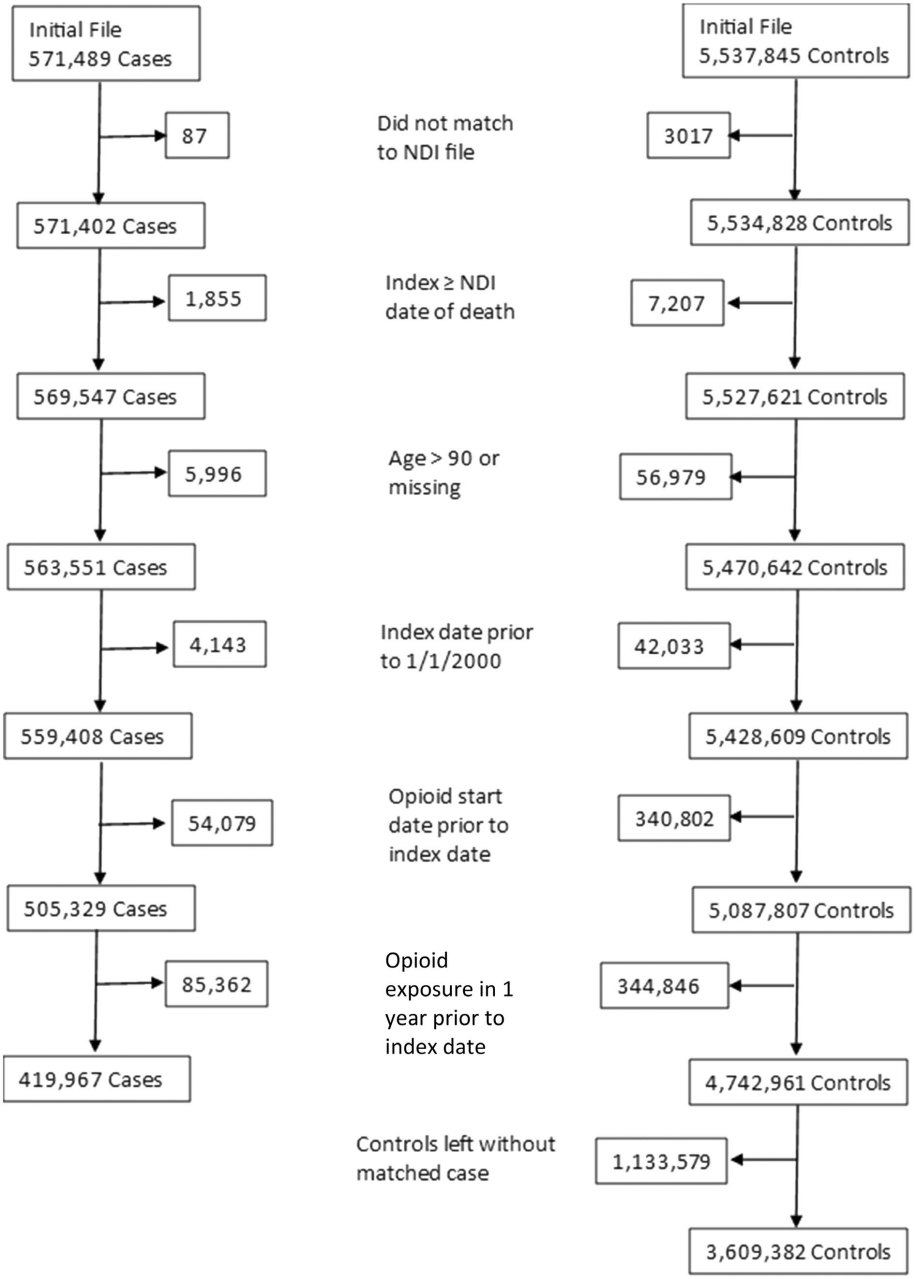
Consolidated Standards of Reporting Trials diagram demonstrating steps and exclusions involved in the development of a matched cohort of patients with gout and controls without gout. NDI, National Death Index.

We identified patients with gout using an administrative algorithm that required patients to have an *International Classification of Diseases, Ninth Revision* (ICD‐9) code of 274.XX from at least two separate encounters at least 30 days apart.^2^ We limited the period for cohort creation up to September 2015 to avoid misclassification from ICD‐10 implementation, which started in October 2015. Each patient with gout was matched with up to 10 patients without gout by birth year, sex, and year of VHA enrollment. Patients without gout (controls) were defined as having no prior gout diagnosis and no previous pharmacy dispensing of urate‐lowering therapy (ULT; allopurinol, febuxostat, probenecid, or pegloticase). Individuals with any opioid prescription in the 365 days before the index date were excluded. A Consolidated Standards of Reporting Trials diagram is provided in Figure 1. Notably, a higher proportion of potential patients with gout (16.9%) than controls (6.8%) were excluded from the analysis due to opioid exposure during the one‐year preindex period.

The index date for patients with gout was defined as the date corresponding to the second diagnostic code, whereas controls were assigned an index date that corresponded to the same date as their matched counterpart with gout. All patients with gout and controls had one year or more of preindex VA follow‐up. Patients were observed from the index date until the earliest of incident opioid exposure leading to long‐term administration, until death, or up to five years following the index date (through July 2020). Follow‐up time was limited to five years because long‐term opioid prescriptions occurring later were more likely to be attributable to causes other than gout. Patients without gout who eventually met the gout algorithm were also censored at the time they fulfilled the algorithm, after which they were matched to controls without gout and contributed to follow‐up time and events as a case. Vital status was determined by linkage with the National Death Index.

### Long‐term opioid exposure

Using a previously validated approach, long‐term opioid exposure was defined as ≥90 cumulative days’ supply from two or more pharmacy dispensing episodes occurring in a six‐month window in the absence of a gap ≥32 days.^14^ Medications considered as opioids in this study included acetaminophen/hydrocodone, buprenorphine, codeine, fentanyl, hydromorphone, methadone, morphine, oxycodone, and tramadol.

### Patient demographics and comorbidities

Covariates were defined using CDW data and a one‐year lookback period. Covariates included age, sex, race and ethnicity, urban or rural residence, body mass index (BMI), smoking status (current, former, never), and comorbidities. As a social determinant of health, race and ethnicity (non‐Hispanic White, non‐Hispanic Black, or other) was included as a covariate given differences in gout burden reported across populations.^15^, ^16^ “Other” race and ethnicity included those reporting American Indian or Alaska Native, Asian, Black Hispanic, multiple races, Native Hawaiian or Pacific Islander, or White Hispanic status. These groups were combined given low numbers of individuals in each category, which resulted in limited power to conduct more granular analyses based on self‐reported race and ethnicity. BMI was calculated from vital signs data using the weight most proximate to the index date and modal height and was categorized as <20, 20 to <25 (referent), 25 to <30, and ≥30. The Rheumatic Disease Comorbidity Index (RDCI) score was calculated as a measure of comorbidity burden.^17^ Comorbid conditions composing the RDCI and those included in the sensitivity analyses were defined by the presence of two or more diagnostic codes^18^ from inpatient or outpatient encounters at any time before or on the index date. Additionally, chronic kidney disease (CKD) was defined using the estimated glomerular filtration rate available from laboratory data and values most proximate to the index date. CKD was classified as stage 1 (>90 mL/min; referent), 2 (60–89 mL/min), 3 (30–59 mL/min), or 4 to 5 (<30 mL/min). Residence was defined using the VA urban–rural indicator variable available in the CDW, with comparisons of individuals defined as urban versus rural or highly rural.^19^

### Gout‐related care factors

To examine associations between measures of gout care and long‐term opioid exposure, serum urate (sU) control and ULT receipt were assessed during each year of follow‐up post index. Briefly, patients with gout with a mean sU level of >7 mg/dL during a one‐year period were considered to have inadequate sU control, and those with a mean sU level of ≤7 mg/dL were considered to have adequate sU control. Patients were considered to have received ULT with at least one episode of pharmacy dispensing of allopurinol, febuxostat, or probenecid during each one‐year block. Missing sU values for each one‐year period were imputed using available values for up to two years prior. Thus, sU control and ULT receipt variables, measured before any long‐term opiate receipt, could change year to year within a patient. As informative measures of gout severity (ie, tophi; flare frequency, intensity, and duration) were not reliably available in the CDW, we modeled rheumatology care as a surrogate of severity.^20^ The requirement of rheumatology consultation was defined as a rheumatology visit occurring during follow‐up with an associated diagnostic code (ICD‐9 274.X or ICD‐10 M10.X). In contrast to other covariates, which were ascertained at baseline or during the at‐risk follow‐up period (ie, sU control/ULT receipt), we considered patients to have satisfied the requirement of rheumatology consultation with any rheumatology encounter occurring during the maximum five‐year follow‐up. This broader time allowance was based on the assumption that a need for subspecialty consultation would serve as a surrogate of gout severity independent of whether it occurred before opioid exposure or in the immediate postexposure period.

### Statistical analyses

Crude incidence rates of long‐term opioid exposure were calculated for those with and without gout. Associations of gout (vs non‐gout) with long‐term prescription opioid exposure were quantified using univariable and then multivariable Cox proportional hazards regression, with the cumulative incidence plotted over time. In addition to accounting for age, sex, and enrollment year through matching, covariates examined in multivariable regression included race and ethnicity, BMI, smoking, urban or rural residence, CKD stage, and RDCI score. To further examine the impact of obesity‐related comorbidities occurring more frequently in gout, the multivariable analysis was repeated after including the presence of baseline diabetes, hypertension, myocardial infarction, and cardiovascular disease (individual components of the RDCI). Univariable and multivariable associations between individual characteristics and long‐term opioid exposure were also examined in separate analyses limited to gout. In addition to the covariates noted, additional covariates in this model included age, sex, enrollment year, a requirement of rheumatology care, and time‐varying measures of sU control and ULT treatment. Allowing for the measurement of sU control and ULT treatment, follow‐up time for this analysis began one‐year post index. To account for diagnoses potentially associated with increased risk of opioid exposure, we performed three separate sensitivity analyses that included the aforementioned covariates in addition to the following baseline conditions: (1) mental health conditions (anxiety, depression, or posttraumatic stress disorder), (2) other chronic pain conditions (chronic headaches, chronic back pain, fibromyalgia, or osteoarthritis), or (3) all of the conditions included in 2 or 3. All analyses were completed using Stata v18 (StataCorp).

## RESULTS

### Participant characteristics

We identified 419,967 patients with gout and 3,609,382 matched controls (Figure 1). The cohort was overwhelmingly male (99%), 59% reported non‐Hispanic White race and ethnicity, and patients had a mean age of approximately 68 years (Table 1). Race and ethnicity, smoking, and CKD status were more likely to be missing for patients without gout compared to those with gout. Patients with gout had higher BMI values and a greater frequency of comorbidity compared to those without gout. Among patients with gout, 8.9% required a rheumatology visit during the up to five‐year follow‐up period. Of the one‐year follow‐up periods assessed in patients with gout, 21% were characterized by inadequate sU control and 46% were characterized by ULT receipt.

**Table 1:** Characteristics of patients with and without gout*

	Without gout (n = 3,609,382)	With gout (n = 419,967)
Age, mean (SD), y	67.6 (11.7)	67.6 (11.6)
Sex, n (%)		
Male	3,579,838 (99)	416,548 (99)
Female	29,544 (1)	3,419 (1)
Race and ethnicity, n (%)		
White, non‐Hispanic	2,131,557 (59)	264,077 (63)
Black, non‐Hispanic	326,407 (9)	59,558 (14)
Other	227,673 (6)	29,659 (7)
Missing	923,745 (26)	66,673 (16)
Residence, n (%)		
Urban	2,336,825 (65)	263,488 (63)
Rural	1,264,075 (35)	155,041 (37)
Missing	8,482 (0)	1,438 (0)
Smoking status, n (%)		
Current	1,102,968 (31)	136,089 (32)
Former	1,218,500 (34)	174,483 (42)
Never	635,606 (18)	83,877 (20)
Missing	652,308 (18)	25,518 (6)
Body mass index, n (%)		
<20	51,072 (2)	3,052 (1)
20 to <25	439,279 (14)	26,852 (6)
25 to <30	1,211,827 (38)	124,227 (30)
30+	1,448,317 (46)	261,198 (63)
RDCI score, mean (SD)	1.15 (1.37)	1.83 (1.35)
RDCI components, n (%)		
Lung disease	264,830 (7)	40,267 (10)
Myocardial infarction,	65,708 (2)	13,444 (3)
Cardiovascular disease	710,233 (20)	141,446 (34)
Stroke	65,444 (2)	11,513 (3)
Hypertension	1,405,801 (39)	296,625 (71)
Diabetes	598,140 (17)	113,448 (27)
Fracture	10,998 (0.3)	1,295 (0.3)
Depression	310,863 (9)	45,496 (11)
Ulcer	81,811 (2)	13,781 (3)
Cancer	268,216 (7)	41,017 (10)
Chronic kidney disease, n (%)		
Stage 1 (>90 mL/min)	633,381 (18)	71,236 (17)
Stage 2 (60–89 mL/min)	1,312,655 (36)	177,803 (42)
Stage 3 (30–59 mL/min)	481,743 (13)	121,557 (29)
Stage 4–5 (<30 mL/min)	47,006 (1)	18,954 (5)
Missing	1,134,597 (31)	30,417 (7)

* All without gout vs with gout comparisons were significant (*P* < 0.001), with the exception of age, sex, and fracture prevalence. RDCI, Rheumatic Disease Comorbidity Index.

### Long‐term opioid exposure in patients with and without gout

**Figure 2:**
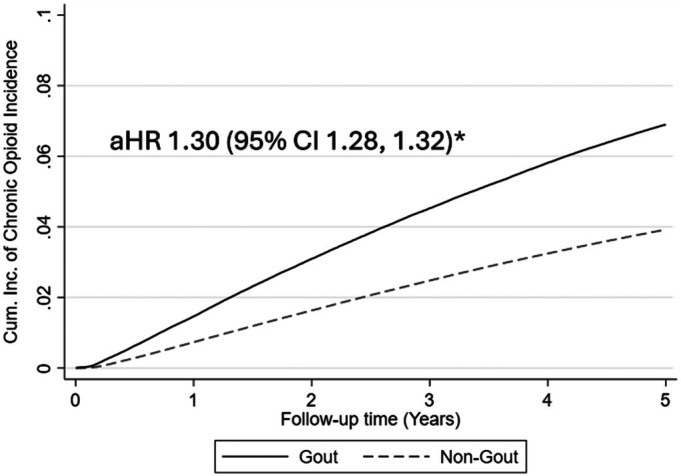
aHR and 95% CI estimated using multivariable Cox proportional hazards regression. Patients with gout and those without gout were matched for age, sex, and year of Veterans Health Administration enrollment. The model includes gout status, race and ethnicity, urban or rural residence, body mass index category, smoking status, Rheumatic Disease Comorbidity Index score, and chronic kidney disease stage. *aHR, adjust hazard ratio; CI, confidence interval; Cum. Inc., cumulative incidence.

Over a follow‐up period of 17.3 million patient‐years (mean follow‐up 4.52 years), 6.9% of patients with gout received long‐term prescription opioids compared to 3.8% of patients without gout (Table 2). The crude incidence (per 1,000 patient‐years) of long‐term opioid exposure was 16.0 (95% confidence interval [CI] 15.8–16.2) for gout versus 8.9 (95% CI 8.9–8.9) in patients without gout, corresponding to an unadjusted hazard ratio of 1.79 (95% CI 1.77–1.82) (Table 2). After accounting for covariates in a multivariable regression model, patients with gout were 30% more likely to receive long‐term prescription opioids during follow‐up (adjusted hazard ratio [aHR] 1.30; 95% CI 1.28–1.32) (Figure 2). The association of gout with long‐term opioid exposure was not significantly changed following the addition of baseline diabetes, hypertension, myocardial infarction, and cardiovascular disease as covariates (aHR 1.32; 95% CI 1.30–1.34; data not shown). The aforementioned estimates (crude incidence in controls and aHR in patients with gout) translate to the occurrence of one additional case of long‐term prescription opioid exposure for every 74 patients with gout seen over a five‐year period. Sensitivity analyses that included baseline mental health conditions and/or other conditions associated with chronic pain as additional covariates did not meaningfully change these results (Supplemental Table 1).

**Table 2:** The frequency, crude incidence rates, and unadjusted/adjusted HRs of long‐term opioid exposure in patients with and without gout*

	Patients initiating long‐term opioids, n (%)	Patient‐years of follow‐up	Crude incidence (95% CI) per 1,000 patient‐years	Unadjusted HR (95% CI)	Adjusted HR (95% CI)^a^
Without gout	137,506 (3.8)	15,453,930	8.9 (8.9–8.9)	Reference	Reference
With gout	28,962 (6.9)	1,809,599	16.0 (15.8–16.2)	1.79 (1.77–1.82)	1.30 (1.28–1.32)

* Patients with gout (n = 419,967) and without gout (n = 3,609,382) were matched on birth year, sex, and Veterans Health Administration enrollment year. CI, confidence interval; HR, hazard ratio.

^a^ Covariates included gout status, race and ethnicity, urban or rural residence, body mass index category, smoking status, Rheumatic Disease Comorbidity Index score, and chronic kidney disease stage (patients and controls matched for age, sex, and year of Veterans Health Administration enrollment).

### Determinants of long‐term opioid exposure in patients with gout

Univariable and multivariable associations between individual characteristics and gout‐related long‐term opioid exposure were examined (Table 3). In a multivariable regression model limited to gout, factors most strongly associated with an increased likelihood of long‐term opioid exposure included female sex (aHR 1.29; 95% CI 1.14–1.46), underweight BMI (aHR 1.32; 95% CI 1.13–1.54), current smoking (aHR 1.62; 95% CI 1.55–1.68), a higher RDCI score (aHR 1.26; 95% CI 1.25–1.27), ULT receipt (aHR 1.30; 95% CI 1.27–1.34), and requirement of rheumatology consultation (aHR 1.60; 95% CI 1.54–1.66). Other factors less strongly associated with an increased risk of long‐term opioid exposure included more recent index year, younger age, non‐Hispanic Black race and ethnicity, rural residence, obese BMI, and former or missing smoking status. Factors associated with a lower risk of long‐term opioid exposure included overweight BMI, the presence of CKD, and evidence of adequate sU control.

**Table 3:** Univariable and multivariable associations of patient characteristics with the receipt of long‐term opioid prescriptions among patients with gout*

Characteristic	Unadjusted HR (95% CI)	Adjusted HR (95% CI)
Demographics		
Age (per 10 y)	0.79 (0.78–0.80)	0.80 (0.79–0.81)
Female sex	1.42 (1.27–1.58)	1.29 (1.14–1.46)
Calendar year (index)	1.00 (1.00–1.00)	1.02 (1.01–1.02)
Race and ethnicity		
Non‐Hispanic White	Referent	Referent
Non‐Hispanic Black	1.25 (1.21–1.29)	1.08 (1.05–1.12)
Other	1.03 (0.99–1.08)	1.00 (0.95–1.06)
Missing	0.69 (0.67–0.72)	0.82 (0.78–0.85)
Residence		
Urban	Referent	Referent
Rural	1.10 (1.08–1.13)	1.12 (1.09–1.15)
Missing	0.94 (0.77–1.15)	1.05 (0.84–1.32)
General health measures		
Body mass index		
Underweight (<20)	1.73 (1.52–1.98)	1.32 (1.13–1.54)
Normal (20–24.9)	Referent	Referent
Overweight (25–29.9)	0.89 (0.84–0.95)	0.88 (0.82–0.94)
Obese (≥30)	1.38 (1.31–1.46)	1.09 (1.02–1.16)
Smoking		
Never	Referent	Referent
Former	1.15 (1.10–1.19)	1.12 (1.08–1.17)
Current	2.04 (1.97–2.11)	1.62 (1.55–1.68)
Missing	1.06 (0.99–1.14)	1.11 (1.02–1.21)
RDCI score	1.23 (1.22–1.24)	1.26 (1.25–1.27)
Chronic kidney disease		
Stage 1	Referent	Referent
Stage 2	0.73 (0.71–0.75)	0.85 (0.82–0.88)
Stage 3	0.75 (0.73–0.78)	0.92 (0.88–0.96)
Stage 4 or 5	0.84 (0.79–0.90)	0.90 (0.83–0.97)
Missing	0.37 (0.35–0.40)	0.61 (0.57–0.66)
Gout‐related factors		
Adequate sU control^a^	0.78 (0.76–0.81)	0.95 (0.93–0.98)
ULT receipt^a^	1.34 (1.31–1.38)	1.30 (1.27–1.34)
Rheumatology encounter	1.97 (1.91–2.04)	1.60 (1.54–1.66)

* All variables listed were included in the model. CI, confidence interval; HR, hazard ratio; RDCI, Rheumatic Disease Comorbidity Index; sU, serum urate; ULT, urate‐lowering therapy.

^a^ Time‐varying covariates.

## DISCUSSION

In this matched cohort study, we found that patients with gout were 30% more likely to be prescribed long‐term opioid therapy when compared to patients without gout, after accounting for other factors influencing receipt. Among patients with gout, a number of factors were associated with a higher likelihood of opioid exposure, including sex, race, being underweight, obesity, comorbidity, lack of sU control, and requiring a rheumatology consultation.

To our knowledge, this is the first large‐scale study to compare rates of long‐term opioid exposure between patients with gout and those without gout, though prior reports have detailed the high prevalence of opioid treatment in the acute setting of gout flare. In a study examining US emergency department records between 2015 and 2017, Dalal and colleagues found that 28% of patients with gout received an opioid at discharge.^7^ In another single‐center study of consecutive patients with gout seen in the emergency department, >50% received an opioid prescription at discharge.^8^ Although we cannot say for certain based on the results from our study that the use of opioids to manage acute pain from gout flares serves as a gateway to long‐term opioid exposure, it is a concern that warrants further investigation. Based on national estimates and the crude incidence observed in this population, our results suggest that as many as 165,000 patients with gout in the United States transition to long‐term opioid use each year.

In addition to acute symptoms from flare, emerging data suggest that gout may be associated with the development of generalized pain hypersensitivity, which could also contribute to higher rates of opioid exposure. One of every five patients with crystal‐proven gout surveyed in a single outpatient rheumatology clinic demonstrated evidence of generalized pain hypersensitivity.^21^ Although opioids offer robust analgesia in the setting of acute pain, evidence now suggests the possibility of opioids promoting paradoxical effects in gout, with these agents demonstrating the capacity to activate the NLRP3 inflammasome, the primary pathologic driver of gout‐related inflammation. NLRP3 activation leads to the activation and release of interleukin‐1β (IL‐1β) and IL‐18, potentially worsening or prolonging the inflammation and pain associated with gouty arthritis.^22^, ^23^ Currently, international guidelines, including those from the American College of Rheumatology and EULAR, do not endorse opioids as a recommended therapy in gout, nor do these explicitly recommend against their use.^24^, ^25^ The findings of our study suggest future guidelines should recommend against opioid use in acute gouty arthritis given a higher risk of long‐term opioid use in this population.

Beyond demonstrating a higher risk in gout, our analyses identified factors among those with gout that appear to influence the receipt of long‐term opioids. Notably, some of the risk factors identified parallel those identified in populations without gout. For example, the temporal increase of long‐term opioid exposure observed between the index years of 2000 and 2015 reflects trends reported from the National Health and Nutrition Survey over a similar time frame.^26^ Likewise, patient characteristics of underweight, obesity, rural residence, and tobacco smoking have all been associated with a higher risk of receiving prescriptions for opioid analgesics in populations without gout.^11^, ^27^, ^28^

Reasons for the higher risk of long‐term opioid exposure among those with greater cumulative comorbidity are unknown. Results from a secondary analysis that included obesity‐related conditions suggest that these comorbidities are unlikely to serve as a meaningful source of confounding. It is possible that the use of prescription opioids in these patients reflects relative contraindications of other analgesics or anti‐inflammatory agents used in gout, a question that warrants additional investigation. Underweight patients were also at higher risk for long‐term opioid exposure for unclear reasons, though these findings are consistent with multiple studies, which have found lower BMI to be associated with higher rates of chronic pain.^29^, ^30^ In contrast to the higher risk associated with overall comorbidity burden, the incidence of long‐term opioid exposure was lower in patients with CKD (a condition not included as a component of the RDCI). Lower use in these patients may reflect conventional guidance calling for marked dose reductions or avoidance of opioids in the context of significant renal dysfunction.^31^

Other risk factors, including female sex and being a person of color, align with disparities previously reported in gout. Compared to men with gout, women with gout are less likely to receive ULT and are more likely to have comorbid CKD and hypertension.^3^, ^32^ Moreover, women with gout are more likely to present with atypical joint involvement and, as a possible result, experience longer diagnostic delays. Both veterans and nonveterans reporting non‐Hispanic Black race and ethnicity are substantially more likely than individuals reporting non‐Hispanic White race to have gout.^33^, ^34^ In addition to a higher prevalence, non‐White patients with gout are also less likely to receive ULT than White patients and are less likely to achieve target sU level goals with treatment.^3^, ^35^ Differences in treatment outcome appear to persist even in the context of protocolized treat‐to‐target ULT and after accounting for other sociodemographic measures and medication adherence.^35^ Given less favorable outcomes reported in these populations, findings herein suggest that previous disparities in gout may be compounded by disproportionate rates of long‐term opioid exposure.

In the absence of validated measures of disease severity in administrative data, we operationalized the requirement for rheumatology consultation as a surrogate of gout severity. In previous work, Edwards and colleagues noted that rheumatologists are far more likely to provide care for patients with tophaceous or uncontrolled gout.^20^ In this previous report, patients with tophaceous or uncontrolled gout were in turn more likely to receive ULT, more frequently accrued emergency department visits, and had higher rates of CKD and heart failure. In addition to observing a higher risk of long‐term opioid exposure with ULT dispensing, perhaps owing to greater gout severity, we identified a modest (albeit statistically significant) association between better sU control and lower risk of long‐term opioid exposure. These results may be impacted by imprecision in defining sU control given the low frequency of regular laboratory assessments and infrequent implementation of treat‐to‐target ULT in real‐world settings such as the VA.^36^ It is possible that the potential for sU control to mitigate long‐term opioid exposure is underestimated in our study, as patients satisfying this definition may have experienced less severe gout and thus garnered less benefit from gout management.

Given restrictions in administrative data available, we were not able to reliably identify either the indication or specialty origin for index prescriptions, results that would inform future efforts to reduce long‐term opioid exposures in this population. Additional limitations include the overwhelmingly male VA population examined, which could limit generalizability. As we were reliant on algorithms leveraging administrative data, misclassification of gout status and long‐term opioid exposure is possible, although we would anticipate such nondifferential misclassification to bias these results toward the null. Notably, the definition of long‐term opioid exposure deployed in our study differs from that recently used in a study of veterans following ambulatory surgery, which required more than 180 days of exposure over a one‐year follow‐up period.^37^ Finally, inherent to observational research, unmeasured confounding is possible.

In conclusion, we found in the VHA that patients with gout were more likely than those without gout to be prescribed opioids, leading to long‐term use, after accounting for other possible determinants of receipt. This study also identified several factors that appear to identify patients with gout at highest risk of receipt, factors that might ultimately be used in targeted efforts to reduce long‐term prescription opioid use in this population.

## AUTHOR CONTRIBUTIONS

All authors contributed to at least one of the following manuscript preparation roles: conceptualization AND/OR methodology, software, investigation, formal analysis, data curation, visualization, and validation AND drafting or reviewing/editing the final draft. As corresponding author, Dr Helget confirms that all authors have provided the final approval of the version to be published and takes responsibility for the affirmations regarding article submission (eg, not under consideration by another journal), the integrity of the data presented, and the statements regarding compliance with institutional review board/Declaration of Helsinki requirements.

## Supporting information


**Disclosure form**:

**Supplemental Table 1** Sensitivity analyses for mental health and chronic pain conditions; adjusted hazard ratio of chronic opioid exposure in patients with and without gout
