# Supplementary material for: Long‐Term Opioids in Gout: A Matched Cohort Study From the Veterans Health Administration
Source: Arthritis Care Res (Hoboken). 2025 Nov 27;78(3):344–51. doi: 10.1002/acr.25622 (PMC12975667; doi:10.1002/acr.25622)
Supplement: Supplementary file 2 — Supplemental Table 1 Sensitivity analyses for mental health and chronic pain conditions; adjusted hazard ratio of chronic opioid exposure in patients with and without gout [file ACR-78-344-s001.docx]

**Supplemental Table 1.** Sensitivity analyses for mental health and chronic pain conditions; adjusted hazard ratio of chronic opioid exposure in patients with and without gout

|  | **Adjusted Hazard Ratio**  **(95% Confidence Interval)**  **Including Mental Health Conditions** | **Adjusted Hazard Ratio**  **(95% Confidence Interval)**  **Including Chronic Pain Conditions** | **Adjusted Hazard Ratio**  **(95% Confidence Interval)**  **Including Chronic Pain and Mental Health Conditions** |
| --- | --- | --- | --- |
| Non-Gout | Reference | Reference | Reference |
| Gout | 1.31 (1.29,1.34) | 1.29 (1.28,1.32) | 1.31 (1.29,1.33) |

*Covariates included in all models shown include: gout status, race/ethnicity, urban/rural residence, body mass index category, smoking status, Rheumatic Disease Comorbidity Index (RDCI) score, and chronic kidney disease stage (cases and controls matched for age, sex, and year of VA enrollment); mental health conditions included depression, anxiety, and post-traumatic stress disorder; chronic pain conditions included chronic headache, chronic back pain, osteoarthritis, and fibromyalgia
